# The Association between Gene Polymorphism of TCF7L2 and Type 2 Diabetes in Chinese Han Population: A Meta-Analysis

**DOI:** 10.1371/journal.pone.0059495

**Published:** 2013-03-19

**Authors:** Haoying Dou, Enting Ma, Liqun Yin, Yinghui Jin, Hongwu Wang

**Affiliations:** 1 Department of Nursing, Tianjin University of Traditional Chinese Medicine, Tianjin, China; 2 Department of Public Education, Tianjin University of Traditional Chinese Medicine, Tianjin, China; 3 Department of Traditional Chinese Medicine, Tianjin University of Traditional Chinese Medicine, Tianjin, China; The Children’s Hospital of Philadelphia, United States of America

## Abstract

In recent years, it has been widely accepted that transcription factor 7-like 2 (TCF7L2) is associated with type 2 diabetes mellitus (T2DM) in multiple ethnic groups, especially its single nucleotide polymorphisms of rs7903146C/T, rs12255372G/T and rs290487T/C. However, the results previously obtained in Chinese Han population are often inconsistent. For clearing this issue, herein we performed meta-analysis based on the reports that can be found to assess the association. In the meta-analysis, Odds ratio (*OR*) and 95% confidence interval (95% *CI*) were calculated with random-effect model or fixed-effect model based on the heterogeneity analysis. The quality of included studies was evaluated by using the Newcastle-Ottawa Scale. The sensitivity analysis was used to confirm the reliability and stability of the meta-analysis. In total, 20 case-control studies with 9122 cases of T2DM and 8017 controls were included. Among these case-control studies, we selected 13 ones on rs7903146 C/T, 5 ones on rs12255372 G/T, 8 ones on rs290487 T/C. The results indicated that rs7903146C/T polymorphism was significantly associated with T2DM (T vs. C, *OR = *1.73, 95% *CI = *1.39–2.16). There was no evidence that rs12255372G/T and rs290487T/C polymorphisms increased T2DM risk (T vs. G, *OR = *1.77, 95% *CI = *0.88–3.56; C vs. T, *OR* = 1.08, 95% *CI = *0.93–1.25). Subgroup analysis of different regions proved the relationship between rs7903146C/T polymorphism and T2DM risk in both the northern and the southern China. The association of rs290487 with T2DM was affected by body mass index, whereas the association of rs7903146 and rs290487 with T2DM was influenced neither by age nor by sex. In conclusion, this study indicated that the rs7903146C/T polymorphism of the TCF7L2 gene had a significant effect on T2DM risk in Chinese Han population, with rs12255372G/T and rs290487T/C polymorphisms showing no significant effect.

## Introduction

One of the most challenging health problems of the twenty-oneth century is type 2 diabetes mellitus (T2DM). It represents a significant disease burden on human beings, both in developed and developing countries [1], and now affects 285 million people all over the world. More importantly, its prevalence is increasing rapidly over the next decade owing to human longevity and surge of obesity in many countries including China [2].

T2DM is a complex metabolic disease that results from the combination of genetic and environmental factors [3]. Grant and colleagues [4] reported on the association of transcription factor 7-like 2 (TCF7L2) polymorphism with T2DM in an Icelandic case-control sample. From then on, the TCF7L2 gene is regarded as one of the most important genes in determining the genetic susceptibility for T2DM in Europeans [4]–[10], West Africans [11], Mexican Americans [12], Southern Asians [13], and Chinese [14]–[17]. However, conflicting results in Chinese Han population [14]–[15], [18], [19] are often reported because of it’s intricately substructure [20].

In this work, we conducted a meta-analysis with large samples based on the representative single nucleotide polymorphisms in Chinese Han population, which have already been much studied. The meta-analysis method using all available studies has proved to be more powerful and can lead to more reliable conclusion in comparison with a single study. Therefore, we employed this method to evaluate the association between TCF7L2 polymorphisms and the T2DM risk in Chinese Han population.

## Materials and Methods

Our study followed the statement of PRISMA for reporting systematic review and meta-analysis [22].

### Search Strategy

In this meta-analysis, we searched literature from the following databanks: China National Knowledge Infrastructure (CNKI), PubMed, Embase, Elsevier, Springer Link, Cochrane Library, and ISI Web of Science. The searching languages included English and Chinese. The employed key words and subject terms were TCF7L2, transcription factor 7-like 2, rs7903146, rs12255372, rs11196218, rs11196205, rs7901695, rs290487, gene polymorphism, diabetes mellitus, type 2, type 2 diabetes mellitus, T2DM, and T2D. Furthermore, the reference lists of thus-obtained eligible studies and relevant review papers were identified by a manual search on this topic. The last research was updated until August 15, 2012.

### Inclusion and Exclusion Criteria

The primary studies included in our meta-analysis should meet the following criteria: (1) the association between TCF7L2 polymorphisms (rs7903146, rs12255372, rs11196218, rs11196205, rs7901695, and rs290487) and T2DM risk in Chinese Han population should be clearly evaluated; (2) the studies must be of case-control study; (3) the papers should clearly describe the diagnoses of T2DM and the sources of cases and controls; (4) the studies should provide original data and sufficient information to estimate odds ratio (*OR*) and corresponding 95% confidence interval (*CI*). The following studies were excluded: (1) those contained duplicate data; (2) those reported in the form of abstract, comment, and review; (3) those except for that using the largest samples, if the identical group published more than one articles based on the same data series.

### Quality Assessment

The Newcastle-Ottawa Scale (NOS) [23] was used to assess the quality of the studies employed in this work. The NOS contains eight items. It is categorized into three dimensions including selection, comparability, and exposure for case-control studies. The selection contains four items, the comparability contains one item, and the exposure contains three items. A star system is used to allow a semi-quantitative assessment of study quality. A study can be awarded a maximum of one star for each numbered item within the selection and exposure categories. A maximum of two stars can be given for comparability. In our meta-analysis, the region was regarded as the most important confounder factor, while the age, sex, and body mass index (BMI) as the second important. The NOS ranges from zero up to nine stars. High quality study should be achieve more than seven stars, medium quality study between four to six stars and poor-quality study less than four stars.

### Data Extraction

For quality control, data were extracted from the studies independently by two of our authors. If the information of genotype distribution was inadequate, we tried to contact the authors of the related paper. The following information was extracted from each article: the last name of the first author, publishing year, region, numbers of cases and controls, numbers of genotypes for cases and controls, and Hardy-Weinberg equilibrium (HWE) in each control group. Any disagreement between our two authors was resolved by consulting the third.

### Ethics Statement

This article was on a meta-analysis. We got the data from previous studies. All the data were analyzed anonymously. We confirmed that all the data did not involve competing interest.

### Statistical Analysis

In this study, we performed both overall and subgroup meta-analysis. In both kinds of the meta-analysis, pooled odds ratios (*OR*) and 95% confidence interval (*CI*) were used to assess the strength of the association between polymorphisms of TCF7L2 and T2DM risk, which were calculated by fixed-effect model or a random effect model chosen based on the heterogeneity test [24]. When the heterogeneity test of *χ^2^*-based *Q*-test reported a *P* value of more than 0.10, we used the fixed-effect model [25]; otherwise a random effect model was performed [26]. Heterogeneity was also assessed by *I^2^* test. The *I^2^* statistic was documented for the percentage of the observed study variability due to heterogeneity rather than chance. (*I^2^* = 0–25%, no heterogeneity; *I^2^* = 25–50%, moderate heterogeneity; *I^2^* = 50–75%, large heterogeneity; *I^2^* = 75–100%, extreme heterogeneity) [27]. In addition, statistical significance of the association between polymorphism and T2DM risk was calculated by *Z*-test. When the *Z*-test reported a *P* < 0.05, there was statistical significance for the association.

The subgroup meta-analysis was employed to reveal the effect of different regions on the overall estimation of the association. The whole region of China was divided into two parts with the Yangtze River as the boundary [28]. The northern region was on the north of the boundary, and the southern on the south. Additionally, other kinds of subgroup analyses were conducted based on the variables like age, sex, and BMI, respectively. As an example for age, the studies showing statistical significance in age (*P*<0.05) between the cases and the controls were assigned to the incomparability subgroup of age, while those with *P*>0.05 were assigned to comparability subgroup. The subgroup analysis was also performed based on Hardy-Weinberg equilibrium (HWE). As HWE is the principal law of the population genetic studies, we calculated *p* value of HWE for the control group of each study based on the Pearson chi-square. If the *P*>0.05, the focus would conform to HWE and the samples of control would be representative.

A sensitivity analysis was carried out to assess the stability of the meta-analysis results. That is to say, by omitting one case–control study at a time, the pooled OR for the remaining studies was computed. If the pooled OR was not changed by the single study, this result would be stability.

In our literature, funnel plots were used to evaluate publication bias. All *P*-values were two-tailed. The Review Manager 5.0 software (2011, Cochrane Collaboration) was used to carry out the meta-analysis.

## Results

### Studies and Data Included in this Meta-analysis

**Figure 1:**
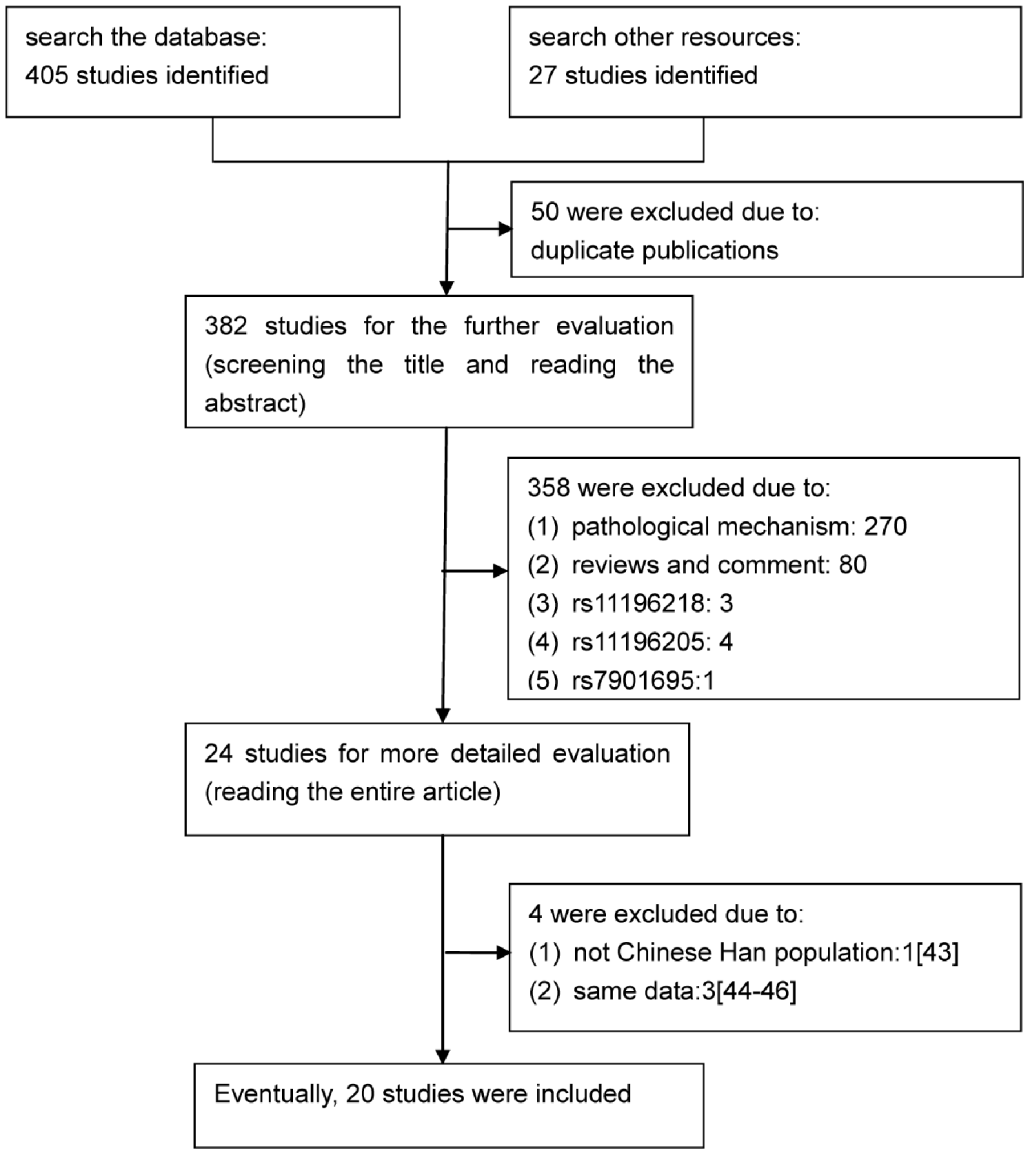
Flow for the selection of eligible studies.

Fig. 1 shows the flow for searching and selecting eligible literature. After removal of the publications of duplicates, reviews, abstracts, and comments, 20 case-control studies [14]–[19], [29]–[42] with 9122 cases of T2DM and 8017 controls were identified for recruitment in the light of the inclusion criteria. All these studies were published from 2007 to 2012.

Table 1 shows 13 eligible studies for rs7903146 with 6427 cases of T2DM and 6114 controls, which were included in this analysis. Table 2 summarizes 5 eligible studies for rs12255372 with 2294 cases of T2DM and 1957 controls. Table 3 shows 8 studies for rs290487 with 2141 cases of T2DM and 1727 controls. The characteristics of all the included studies are also listed in the tables, such as source of controls, HWE, and comparability of cases and controls. Table 4 summarizes the quality of all the included studies assessed by NOS. Most studies were of high quality in terms of selection and exposure. However, as for comparability, the quality was relativity low, since most of the cases were not quite comparable with the controls on age, sex, and BMI.

**Table 1:** The basic characteristics of the included studies for rs7903146.

First author	Publishing year	Region	Source of controls	Sample sizes		Case			Control			HWE	Comparability
				Case	Control	TT	TC	CC	TT	TC	CC		
Chang	2007	Taiwan	Hospital-based	760	760	1	35	724	0	44	716	Yes	Age**,sex**,BMI**
Ng	2007	HongKong	community-based and hospital staff	433	419	1	24	408	0	20	399	Yes	Age*, sex*, BMI**
Zeng	2007	Chengdu	Hospital staff	92	80	0	8	84	0	2	78	Yes	Age* and BMI**
Ren	2008	Beijing	Hospital-based	500	500	2	41	438	2	26	463	No	Age**,sex**,BMI**
Wang	2008	Chongqing	Hospital-based	446	303	8	67	371	1	24	278	Yes	Age**,sex*, BMI**
Lou	2009	Jiangsu	N/D	682	551	0	49	633	0	25	526	Yes	Age*,sex**, BMI**
Wen	2010	Shanghai	N/D	1165	1136	0	120	1045	3	68	1065	No	Age*, sex*, BMI*
Zhang	2010	Hengyang	Hospital-based	236	218	0	23	213	0	15	203	Yes	Age**,sex*, BMI**
Lin	2010	Chengdu	Hospital-based	1529	1439	3	178	1348	4	107	1328	Yes	Age**,sex*, BMI**
Zhao	2011	Qingdao	Hospital-based	99	114	0	11	88	0	4	110	Yes	Age*, sex*, BMI**
Zheng	2011	Chongqing	Hospital-based	227	152	1	24	202	0	13	139	Yes	Age*,sex**, BMI**
Chen	2011	Hubei	Hospital-based	258	239	9	57	192	4	33	202	Yes	Age**,sex*, BMI**
Zhang	2012	Shenyang	N/D	202	203	0	29	173	0	12	191	Yes	Age*, sex*, BMI**

Abbreviations: HWE, Hardy–Weinberg equilibrium; Yes, the genotype distribution meet the HWE in control group; No, the genotype distribution not meet HWE in control group; community-based and hospital staff : subjects who were enrolled from community and hospital staff; Hospital-based: subjects who were enrolled from health check conducted in hospital; N/D: not description; *: P>0.05; **: P<0.05.

**Table 2:** The basic characteristics of the included studies for rs12255372.

First author	Publishing year	Region	Source of controls	Sample sizes		Case		Control		HWE	Comparability
				Case	Control	T	G	T	G		
Chang	2007	Taiwan	Hospital-based	760	760	9	1511	6	1514	Yes	Age**,sex**,BMI**
Ren	2008	Beijing	Hospital-based	500	500	9	989	9	989	No	Age**,sex**,BMI**
Wang	2008	Chongqing	Hospital-based	446	303	15	877	8	598	Yes	Age**,sex*, BMI**
Fan	2009	Tianjin	Hospital-based	352	176	65	639	5	347	Yes	Age*,sex*, BMI**
Zhang	2010	Hengyang	Hospital-based	236	218	17	455	12	424	Yes	Age**,sex*, BMI**

Abbreviations: HWE, Hardy–Weinberg equilibrium; Yes, the genotype distribution meet the HWE in control group; No, the genotype distribution not meet HWE in control group.

Hospital-based: subjects who were enrolled from health check conducted in hospital; *: P>0.05; **: P<0.05.

**Table 3:** The basic characteristics of the included studies for rs290487.

First author	Publishing year	Region	Source of controls	Sample sizes		Case		Control		HWE	Comparability
				Case	Control	C	T	C	T		
Chang	2007	Taiwan	Hospital-based	760	760	635	885	552	968	Yes	Age**,sex**,BMI**
Ren	2008	Beijing	Hospital-based	500	500	391	609	352	648	No	Age**,sex**,BMI**
Qiao	2012	Harbin	Hospital-based	700	570	526	866	466	648	Yes	Age*, sex*, BMI*
Yu	2010	Hunan	N/D	295	188	217	373	143	233	Yes	Age*, sex*, BMI**
Zhang^1^	2008	Jinan	N/D	100	100	79	121	72	128	Yes	Age*, sex*, BMI*
Zhang^2^	2008	Hunan	Hospital-based	536	475	380	664	374	552	Yes	Age*, sex**, BMI*
Zhu	2011	Anhui	Hospital-based	300	300	248	352	205	395	Yes	Age*, sex*, BMI*
Zou	2009	Yunnan	N/D	210	94	139	261	56	132	Yes	Age*, sex*, BMI^N/D^

Abbreviations: HWE, Hardy–Weinberg equilibrium; Yes, the genotype distribution meet the HWE in control group; No, the genotype distribution not meet HWE in control group.

Zhang^1^ : ZhangYong; Zhang^2^ : ZhangYing; Hospital-based: subjects who were enrolled from health check conducted in hospital; N/D: not description; *: P>0.05; **: P<0.05.

**Table 4:** Quality assessment for all the included studies.

First author	Publishing year	Selection	Comparability	Exposure
Chang	2007	☆☆☆	☆	☆☆☆
Ng	2007	☆☆☆	☆	☆
Zeng	2007	☆☆☆		☆☆
Ren	2008	☆☆	☆	☆☆
Wang	2008	☆☆☆	☆	☆☆
Lou	2009	☆☆		☆☆
Fan	2009	☆☆☆☆	☆☆	☆☆☆
Wen	2010	☆☆	☆	☆☆☆
Zhang	2010	☆☆☆☆	☆	☆
Lin	2010	☆☆	☆	☆☆
Zhao	2011	☆☆☆☆	☆☆	☆
Zheng	2011	☆☆☆☆	☆	☆☆
Chen	2011	☆☆☆	☆	☆☆☆
Zhang	2012	☆☆	☆	☆☆
Qiao	2012	☆☆☆	☆☆	☆
Yu	2010	☆☆	☆	☆☆
Zhang^1^	2008	☆☆☆	☆	☆☆
Zhang^2^	2008	☆☆	☆	☆☆
Zhu	2011	☆☆☆☆	☆☆	☆☆
Zou	2009	☆☆	☆	☆☆

Zhang^1^ : Yong Zhang; Zhang^2^ : Ying Zhang.

### Association between rs7903146C/T Polymorphism and T2DM Risk

**Figure 2:**
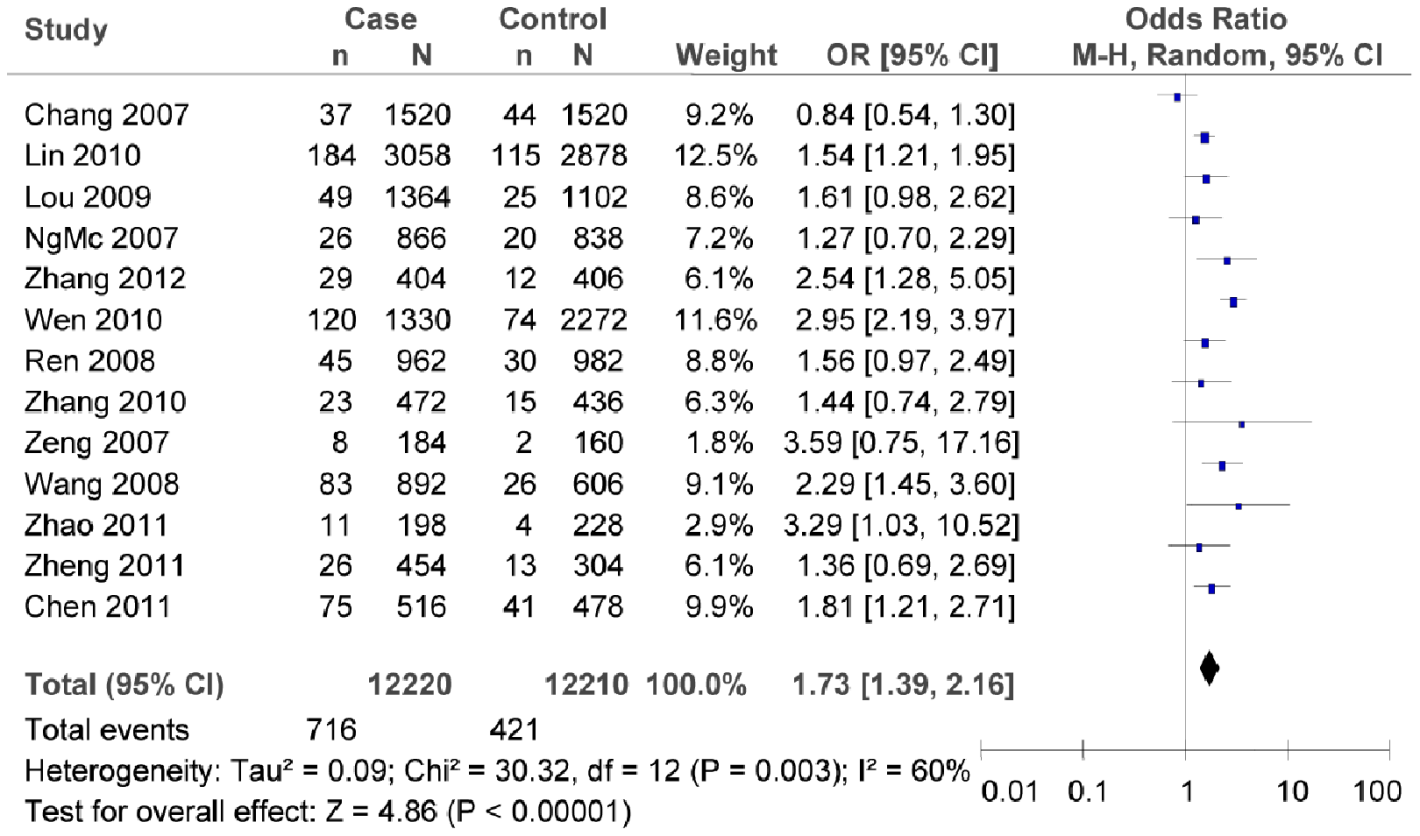
Forest plot of the association between rs7903146C/T polymorphism and T2DM risk (overall meta-analysis T vs. C). n indicates the total number of T allele, and N indicates the total number of T allele plus C allele.

There was significant heterogeneity among the studies of rs7903146C/T in the overall meta-analysis. Therefore, the random effect model was employed to assess the association between rs7903146C/T polymorphism and T2DM risk. The evaluation result indicated that rs7903146C/T polymorphism was associated with T2DM risk (as shown in Fig. 2, T vs. C: *OR* = 1.73, 95% *CI = *1.39–2.16, *P*<0.00001; heterogeneity test *χ* ^2^ = 30.32, *P = *0.003, *I* ^2^ = 60%).

**Figure 3:**
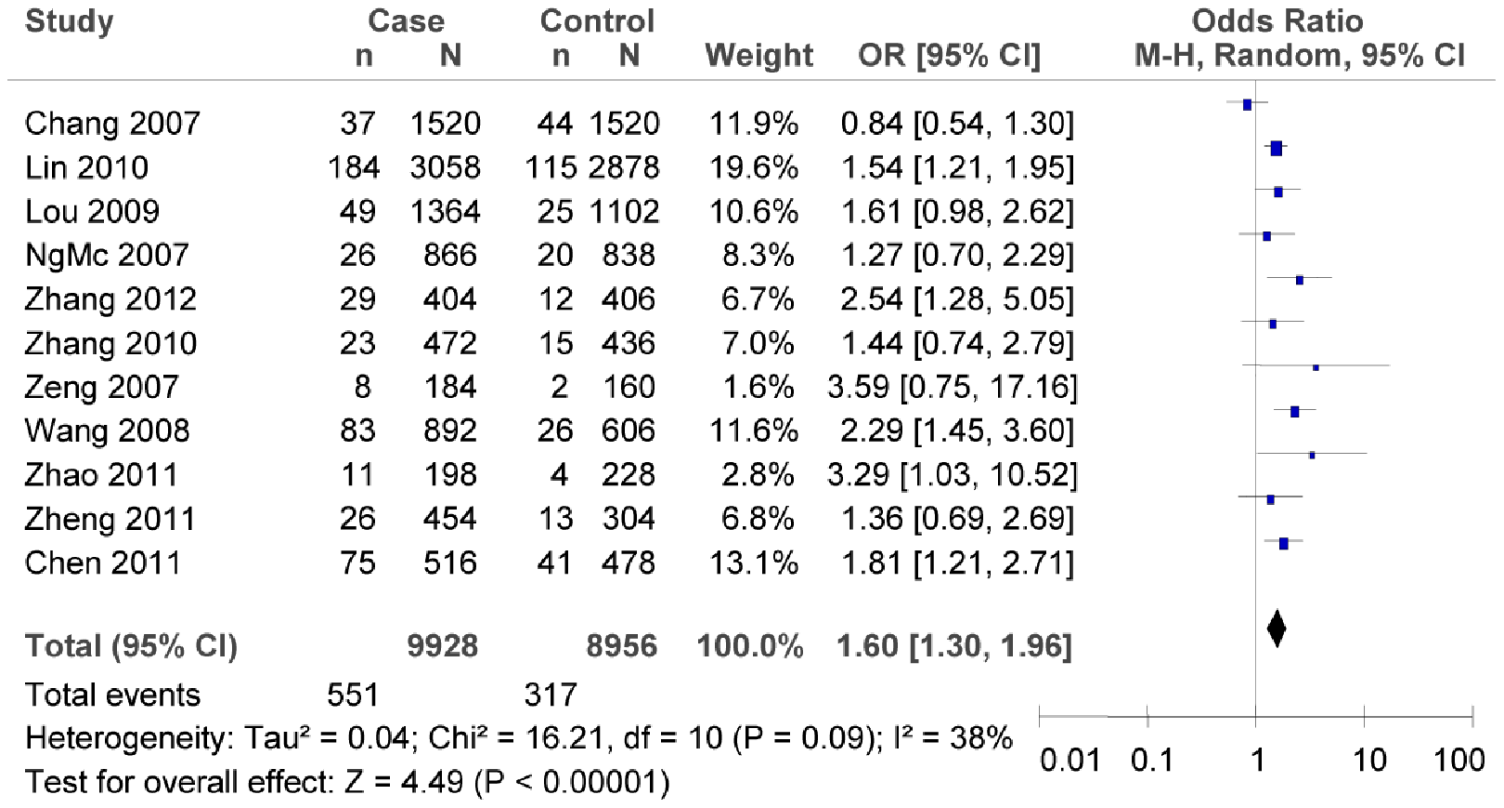
Forest plot of the association between rs7903146C/T polymorphism and T2DM risk (subgroup analyses for the HWE in the control groups: T vs. C). n indicates the total number of T allele, and N indicates the total number of T allele plus C allele.

**Figure 4:**
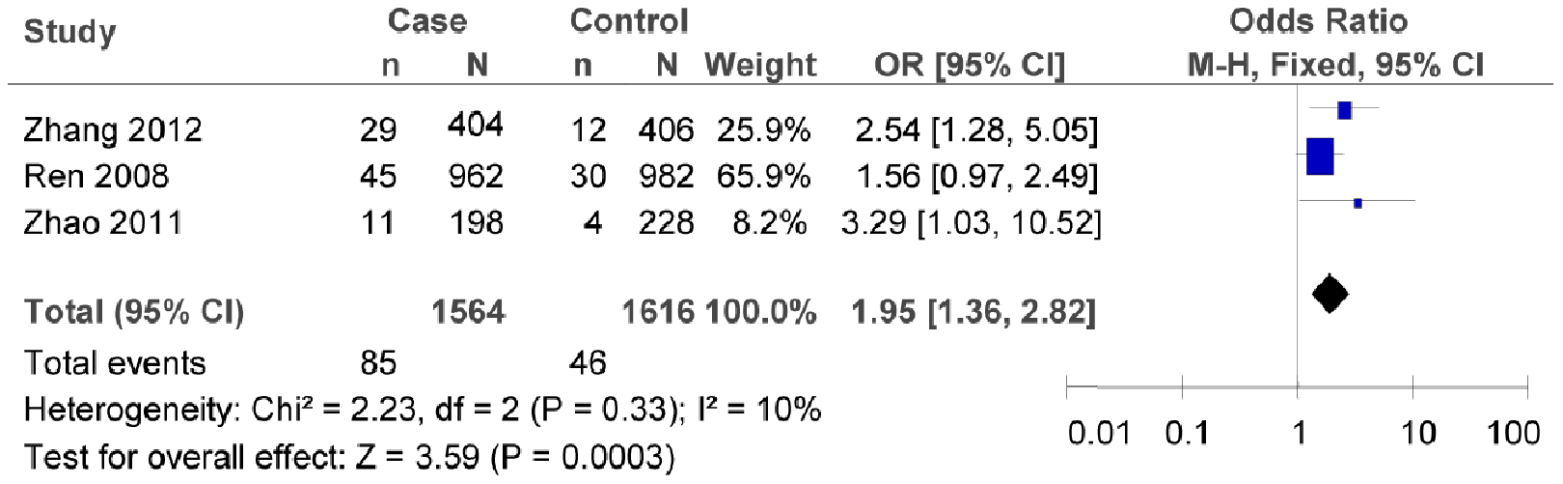
Forest plot of the association between rs7903146C/T polymorphism and T2DM risk (subgroup analyses for the northern: T vs. C). n indicates the total number of T allele, and N indicates the total number of T allele plus C allele.

**Figure 5:**
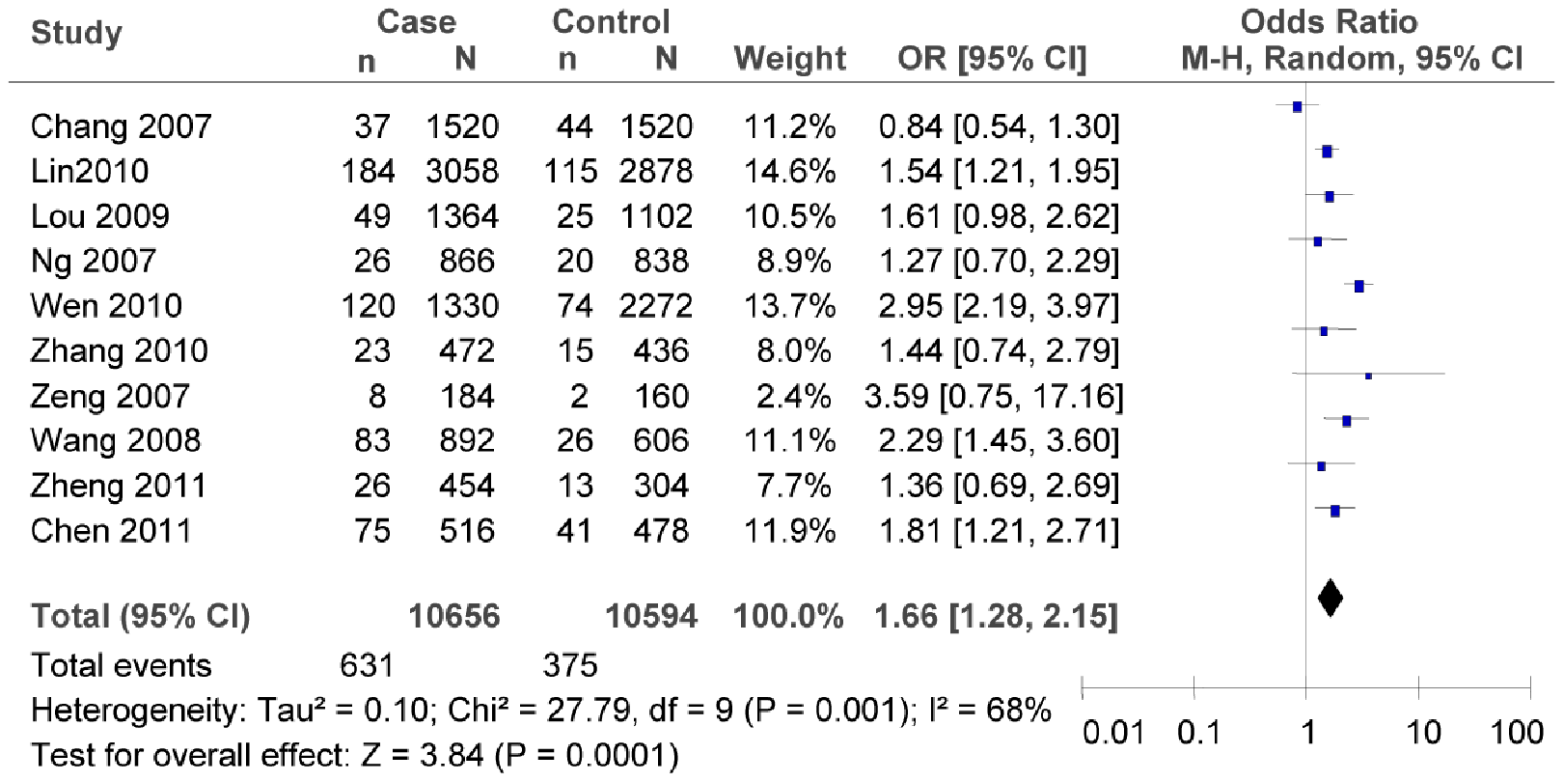
Forest plot of the association between rs7903146C/T polymorphism and T2DM risk (subgroup analyses for the southern: T vs. C). n indicates the total number of T allele, and N indicates the total number of T allele plus C allele.

The subgroup meta-analysis of the studies which conformed with HWE in the control groups showed that there was association between rs7903146C/T polymorphism and T2DM risk (as shown in Fig. 3, T vs. C: *OR* = 1.60, 95% *CI = *1.30–1.96, *P*<0.00001; heterogeneity test *χ* ^2^ = 16.21, *P = *0.09, *I^2^* = 38%). The subgroup meta-analysis of different regions also confirmed that there was association between rs7903146C/T polymorphism and T2DM risk in both the northern and the southern China (as shown in Fig. 4, in the northern China: T vs. C: *OR* = 1.95, 95% *CI* = 1.36–2.82, *P = *0.0003; heterogeneity test *χ* ^2^ = 2.23, *P* = 0.33, *I^2^* = 10%; as shown in Fig. 5, in the southern China, T vs. C: *OR* = 1.66, 95% *CI = *1.28–2.15, *P* = 0.0001; heterogeneity test *χ* ^2^ = 27.79, *P = *0.001, *I^2^* = 68%).

By the subgroup meta-analysis of the studies based on the confounder factors like age and sex, it was illustrated that the association between rs7903146C/T polymorphism and T2DM was not affected by these two factors (the subgroup of age comparability: *OR* = 2.04, 95% *CI* = 1.46–2.84, *P*<0.0001; heterogeneity test *χ* ^2^ = 11.56, *P* = 0.07, *I^2^* = 48%; the subgroup of age incomparability: *OR* = 1.52, 95% *CI* = 1.18–1.96, *P* = 0.001; heterogeneity test *χ* ^2^ = 10.88, *P* = 0.05, *I^2^* = 54%; the subgroup of sex comparability: *OR = *1.97, 95% *CI = *1.54–2.52, *P*<0.00001; heterogeneity test *χ* ^2^ = 15.97, *P* = 0.03, *I^2^* = 56%; the subgroup of sex incomparability: *OR = *1.34, 95% *CI* = 0.95–1.88, *P* = 0.03; heterogeneity test *χ* ^2^ = 6.72, *P* = 0.15, *I^2^* = 40%.). The effect of the BMI on the association was not examined since there was only one study based on the comparability of BMI.

### Association between rs12255372G/T Polymorphism and T2DM Risk

**Figure 6:**
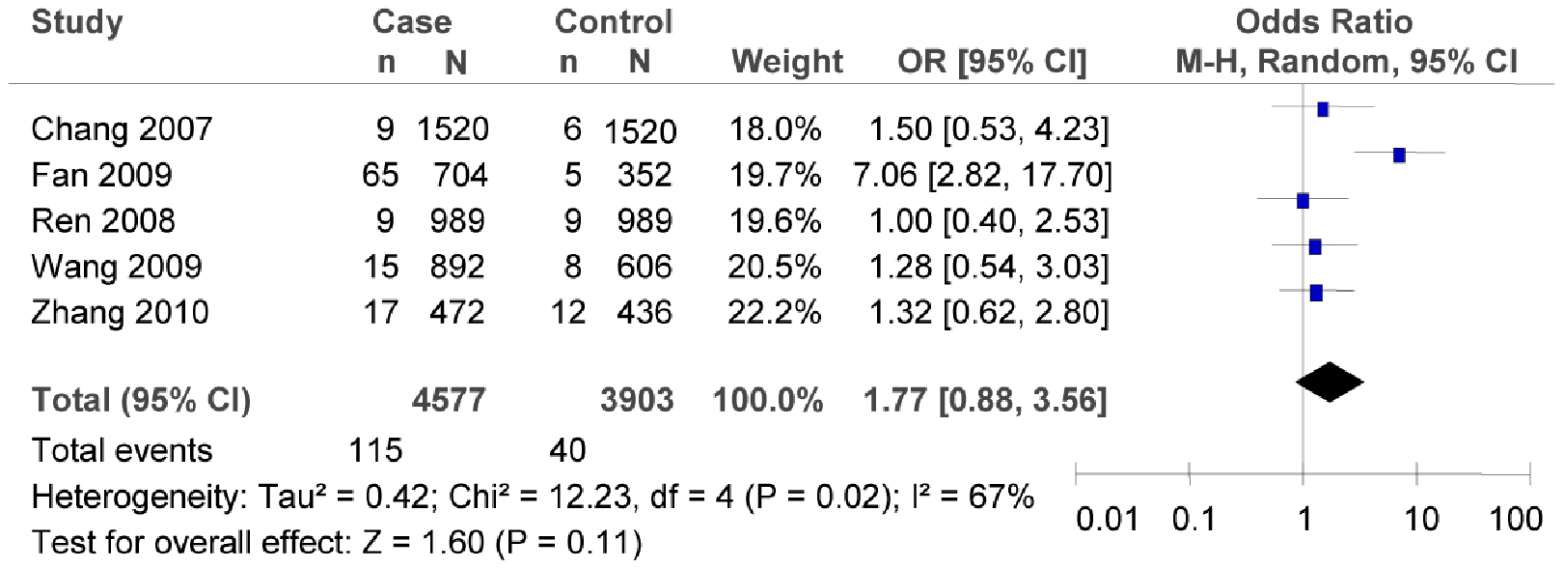
Forest plot of the association between rs12255372G/T polymorphism and T2DM risk (overall meta-analysis T vs. G). n indicates the total number of T allele, and N indicates the total number of T allele plus G allele.

Significant heterogeneity was found among the studies of rs12255372G/T in the overall meta-analysis. Thus, the random effect model was used to evaluate the association between rs12255372G/T polymorphism and T2DM risk. The evaluation result indicated that rs12255372G/T polymorphism was not associated with T2DM risk (as shown in Fig. 6, T vs. G: *OR* = 1.77, 95% *CI* = 0.88–3.56, *P* = 0.11; heterogeneity test *χ* ^2^ = 12.23, *P = *0.02, *I^2^* = 67%).

**Figure 7:**
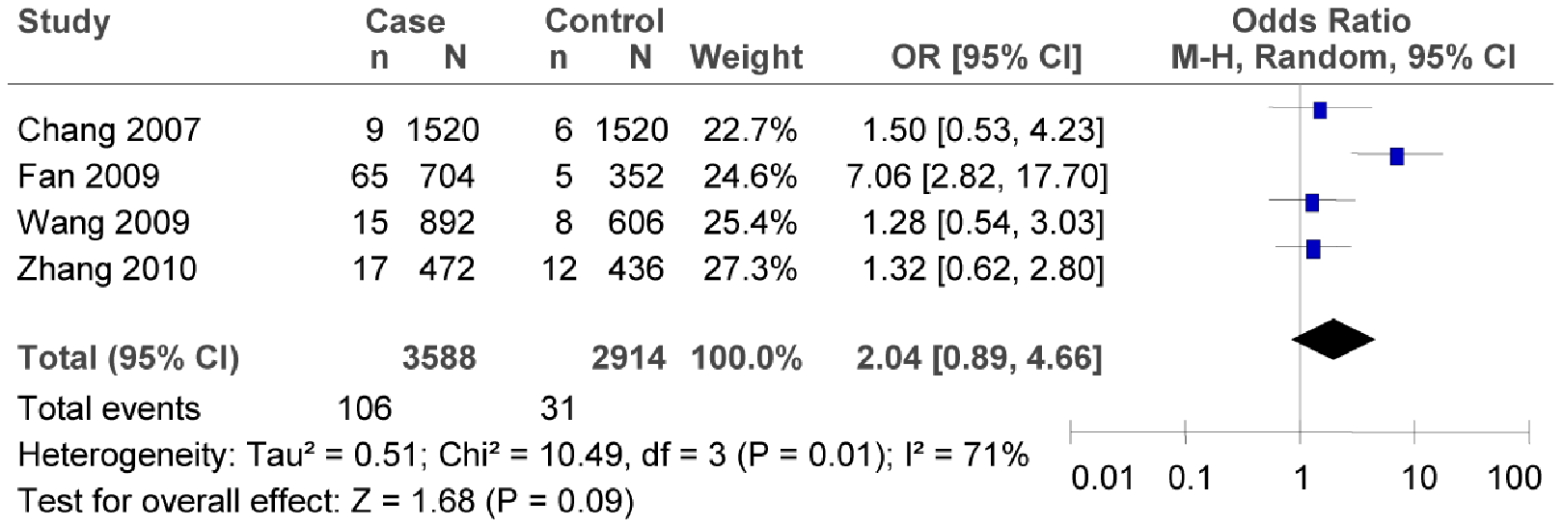
Forest plot of the association between rs12255372G/T polymorphism and T2DM risk (subgroup analyses for the HWE in the control groups: T vs. G). n indicates the total number of T allele, and N indicates the total number of T allele plus G allele.

**Figure 8:**
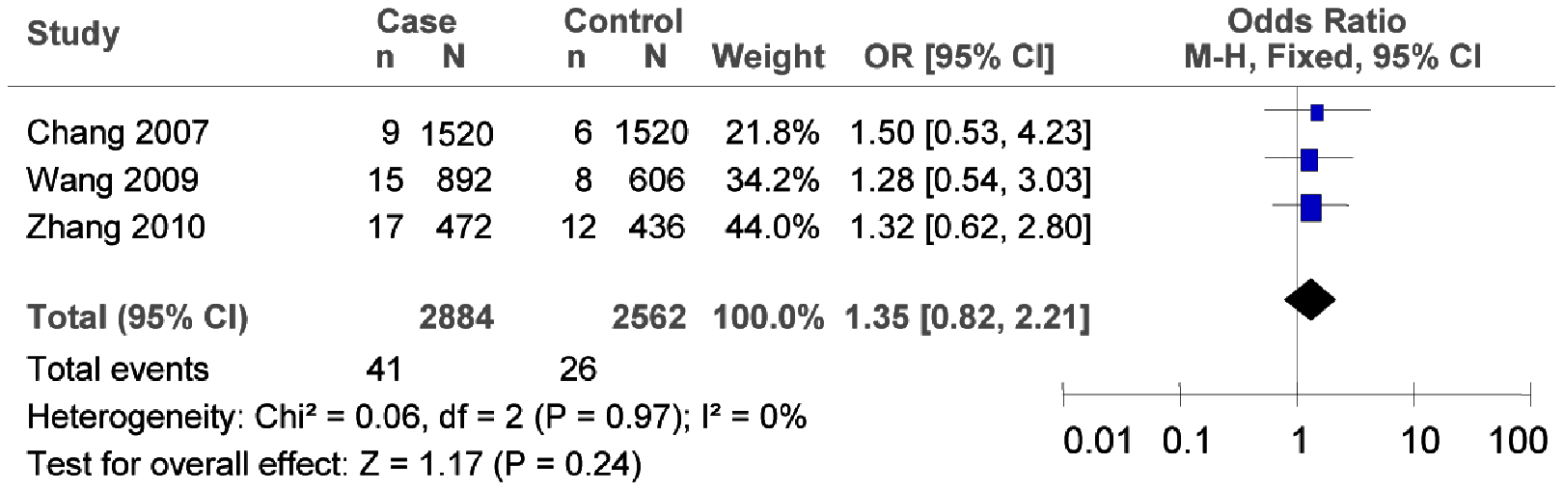
Forest plot of the association between rs12255372G/T polymorphism and T2DM risk (subgroup analyses for the southern China: T vs. G). n indicates the total number of T allele, and N indicates the total number of T allele plus G allele.

The subgroup meta-analysis of the studies which conformed with HWE in the control groups showed that there was no association between 12255372G/T polymorphism and T2DM risk (as shown in Fig. 7, T vs. G: *OR* = 2.04, 95% *CI* = 0.89–4.66, *P* = 0.09; heterogeneity test *χ* ^2^ = 10.49, *P* = 0.01, *I^2^* = 71%). The subgroup meta-analysis also demonstrated that there was no association between 12255372G/T polymorphism and T2DM risk in the southern China (as shown in Fig. 8, in the southern China, T vs. G: *OR* = 1.35, 95% *CI* = 0.82–2.21, *P* = 0.24; heterogeneity test *χ* ^2^ = 0.06, *P* = 0.97, *I^2^* = 0%). We did not evaluate such association for the subgroup of the northern China since there were not sufficient data.

The subgroups analysis of the studies based on age, sex, and BMI was not carried out due to the extreme heterogeneity (*I^2^* = 80%) of sex comparability subgroup and insufficient studies with regard to the age and BMI comparability.

### Association between rs290487T/C Polymorphism and T2DM Risk

**Figure 9:**
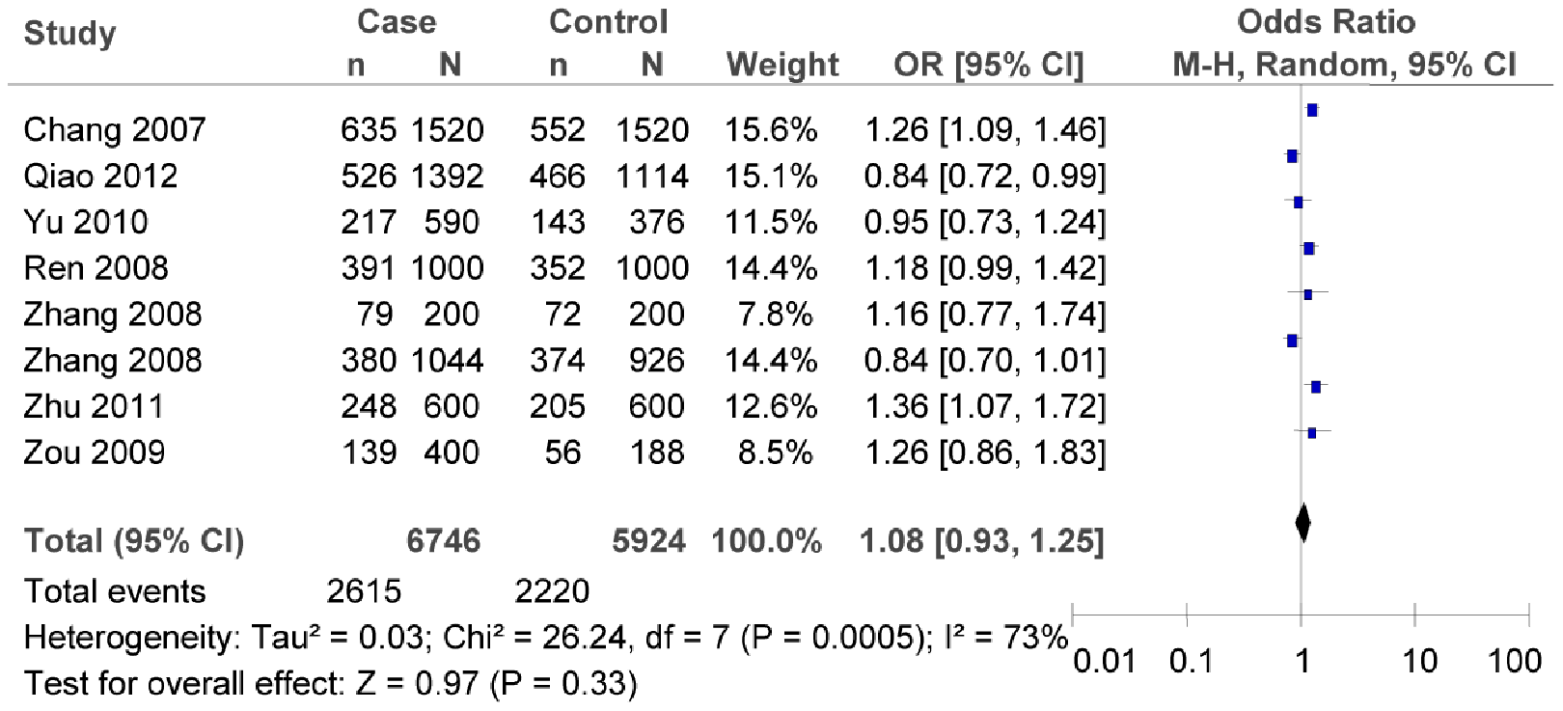
Forest plot of the association between rs290487T/C polymorphism and T2DM risk (overall meta-analysis C vs. T). n indicates the total number of C allele, and N indicates the total number of C allele plus G allele.

Significant heterogeneity was also observed among the studies of rs290487T/C in the overall meta-analysis. Hence the random effect model was chosen to illustrate the association between rs290487T/C polymorphism and T2DM risk. The result confirmed that rs290487C/T polymorphism was not associated with T2DM risk (as shown in Fig. 9, C vs. T: *OR* = 1.08, 95% *CI* = 0.93–1.25, *P* = 0.33; heterogeneity test *χ* ^2^ = 26.24, *P = *0.0005, *I^2^* = 73%).

**Figure 10:**
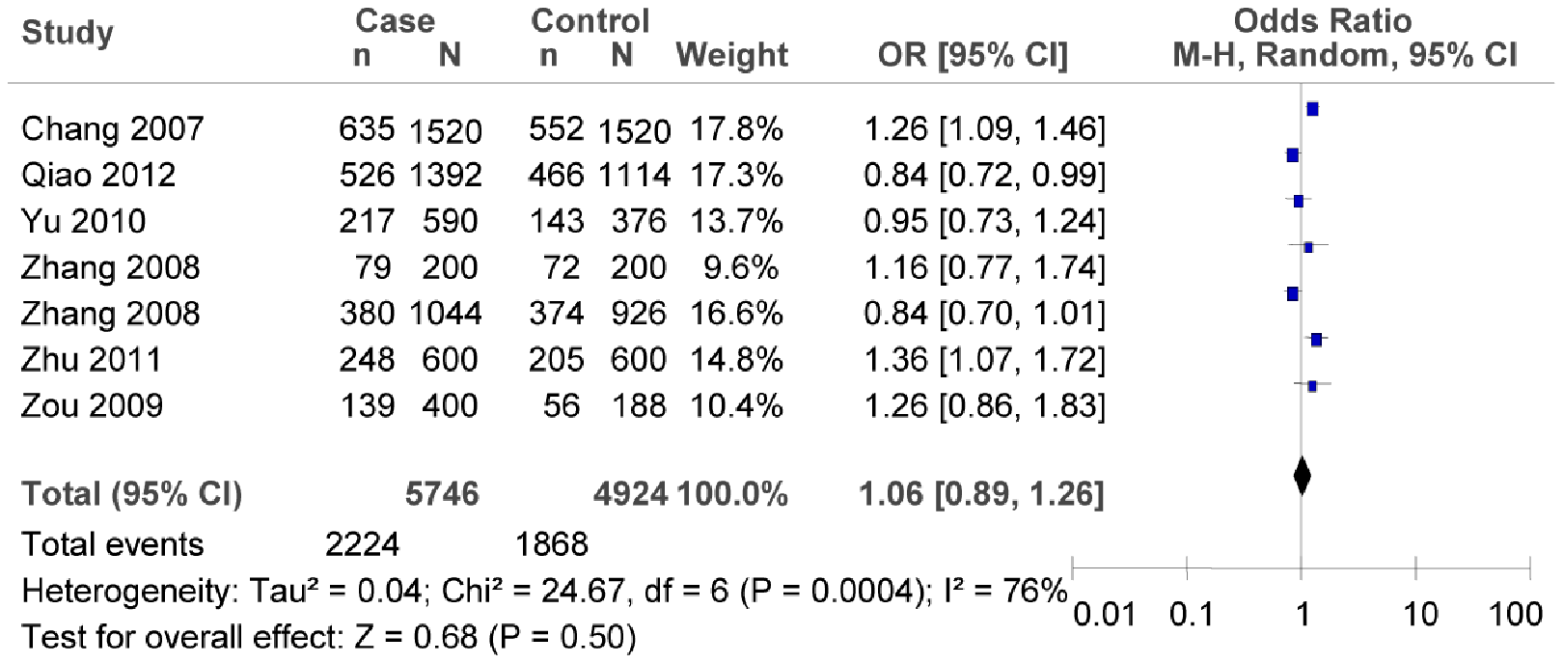
Forest plot of the association between rs290487 T/C polymorphism and T2DM risk (subgroup analyses for the HWE in the control groups: C vs. T). n indicates the total number of C allele, and N indicates the total number of C allele plus G allele.

**Figure 11:**
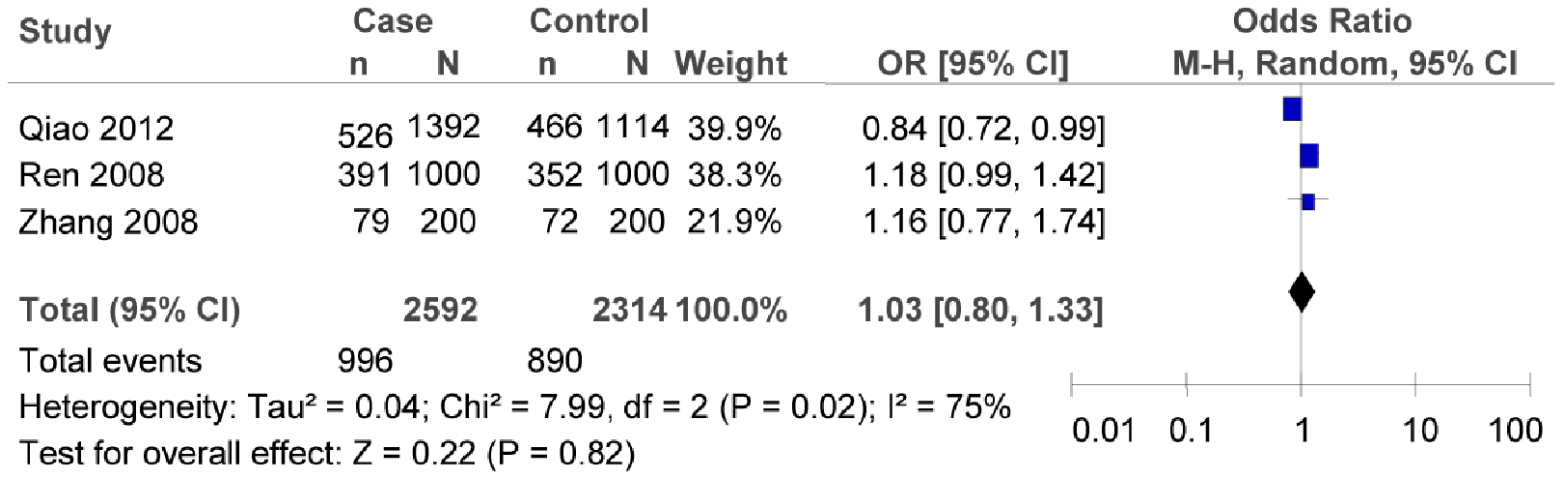
Forest plot of the association between rs290487 T/C polymorphism and T2DM risk (subgroup analyses for the northern China: C vs. T). n indicates the total number of C allele, and N indicates the total number of C allele plus G allele.

**Figure 12:**
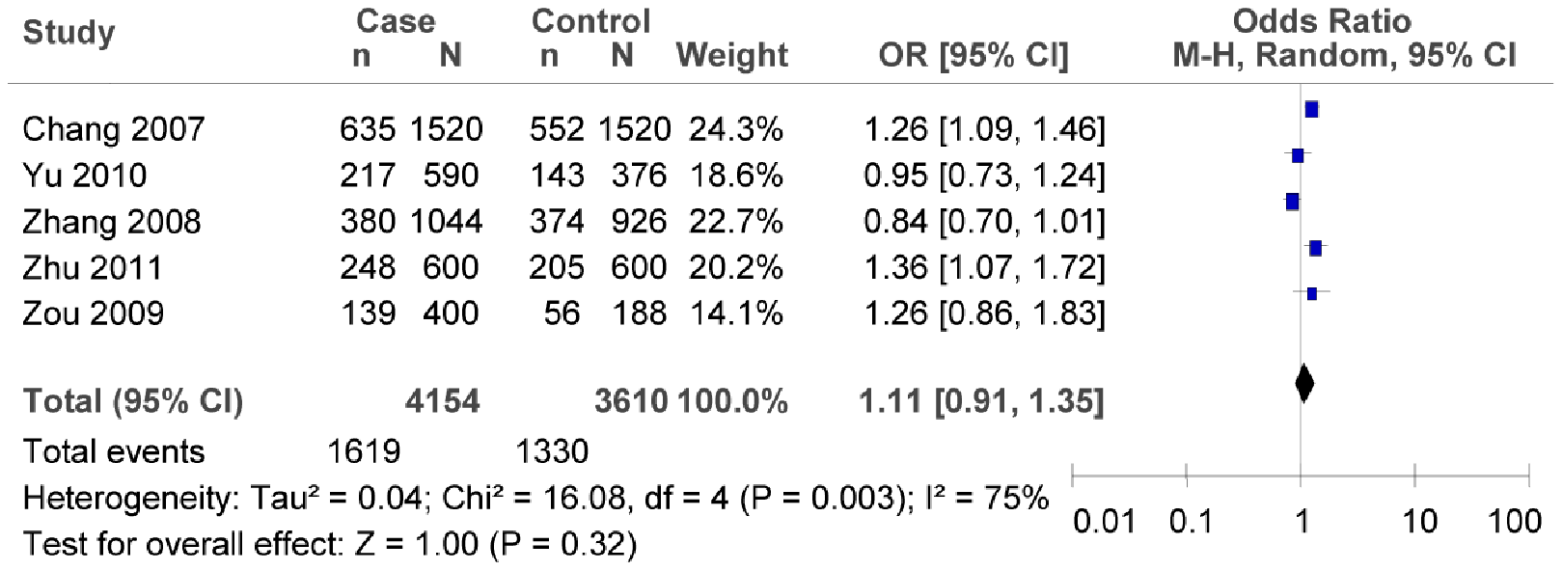
Forest plot of the association between rs290487 T/C polymorphism and T2DM risk (subgroup analyses for the southern China: C vs. T). n indicates the total number of C allele, and N indicates the total number of C allele plus G allele.

Based on the subgroup analyses with HWE in the control groups, we did not find any association between rs290487T/C polymorphism and T2DM risk (as shown in Fig. 10 *OR* = 1.06, 95% *CI* = 0.89–1.26, *P* = 0.50; heterogeneity test *χ* ^2^ = 24.67, *P* = 0.0004, *I^2^* = 76%). Both the subgroup meta-analysis of the southern or the northern China indicated that there was no significant association between rs290487T/C polymorphism and T2DM risk (as shown in Fig. 11, in the northern China:*OR = *1.03, 95% *CI* = 0.80–1.33, *P* = 0.82; heterogeneity test *χ* ^2^ = 7.99, *P* = 0.02, *I^2^* = 75%; as shown in Fig. 12, in the southern China: *OR* = 1.11, 95% *CI* = 0.91–1.35, *P* = 0.32; heterogeneity test *χ* ^2^ = 16.08, *P* = 0.003, *I^2^* = 75%).

The subgroup meta-analysis of the studies based on age illustrated that there was no significant association between rs290487T/C polymorphism and T2DM risk (the subgroup of age comparability: *OR* = 1.02, 95% *CI* = 0.85–1.22, *P* = 0.83; heterogeneity test *χ* ^2^ = 15.56, *P* = 0.008, *I^2^* = 68%). The subgroup analysis of age incomparability was not conducted because of insufficiency studies.

The subgroup meta-analysis of the studies based on sex illustrated that this association was not affected by sex (the subgroup of sex comparability: *OR* = 1.07, 95% *CI* = 0.87–1.33, *P* = 0.51; heterogeneity test *χ* ^2^ = 12.83, *P* = 0.01, *I^2^* = 69%; the subgroup of sex incomparability: *OR* = 1.08, 95% *CI* = 0.85–1.58, *P* = 0.51; heterogeneity test *χ* ^2^ = 11.94, *P* = 0.003, *I^2^ = *83%). On the other hand, the subgroup meta-analysis based on BMI showed that the association was affected by this cofounder factor (the subgroup of BMI comparability: *OR* = 1.01, 95% *CI = *0.79–1.28, *P = *0.96; heterogeneity test *χ* ^2 = ^13.51, *P* = 0.004, *I^2^* = 78%; the subgroup of BMI incomparability: *OR = *1.19, 95% *CI* = 1.07–1.31, *P* = 0.0009; heterogeneity test *χ* ^2^ = 3.42, *P* = 0.33, *I^2^* = 12%).

### Sensitivity Analysis

By omitting one case–control study at a time and computing the pooled ORs for the remaining studies, we found that no single study could change the pooled results (as shown in Table 5, Table 6, and Table 7). That is to say, our results of meta-analysis were very reliable.

**Table 5:** The result of sensitivity analysis with each study omitted for rs7903146C/T.

Study omitted	OR	95% CI	P
Chang	1.86	1.54–2.25	<0.00001
Lin	1.76	1.36–2.27	<0.0001
Lou	1.74	1.37–2.22	<0.00001
NgMc	1.77	1.41–2.24	<0.00001
Zhang^1^	1.69	1.34–2.13	<0.00001
Wen.	1.58	1.38–1.81	<0.00001
Ren	1.75	1.37–2.23	<0.00001
Zhang^2^	1.75	1.39–2.22	<0.00001
Zeng	1.71	1.36–2.14	<0.00001
Wang	1.68	1.33–2.13	<0.0001
Zhao	1.70	1.36–2.13	<0.0001
Zheng	1.76	1.39–2.22	<0.00001
Chen	1.72	1.35–2.20	<0.0001

Abbreviations: OR, odds ratio; CI, confidence interval.

Zhang^1^: XL Zhang; Zhang^2^: L Zhang.

**Table 6:** The result of sensitivity analysis for rs12255372G/T.

Study omitted	OR	95% CI	P
Chang.	1.84	0.77–4.38	0.17
Fan.	1.26	0.81–1.95	0.30
Ren.	2.04	0.89–4.66	0.09
Wang.	1.93	0.79–4.68	0.15
Zhang.	1.95	0.77–4.80	0.16

Abbreviations: OR, odds ratio; CI, confidence interval.

**Table 7:** The result of sensitivity analysis for rs290487 T/C.

Study omitted	OR	95% CI	P
Chang	1.04	0.89–1.22	0.59
Qiao	1.12	0.97–1.30	0.11
Ren	1.06	0.89–1.26	0.50
Yu	1.10	0.93–1.29	0.28
Zhang^1^	1.07	0.91–1.26	0.41
Zhang^2^	1.12	0.96–1.30	0.14
Zhu	1.04	0.89–1.21	0.62
Zou	1.06	0.90–1.24	0.47

Abbreviations: OR, odds ratio; CI, confidence interval.

Zhang^1^ : Yong Zhang; Zhang^2^ : Ying Zhang.

**Figure 13:**
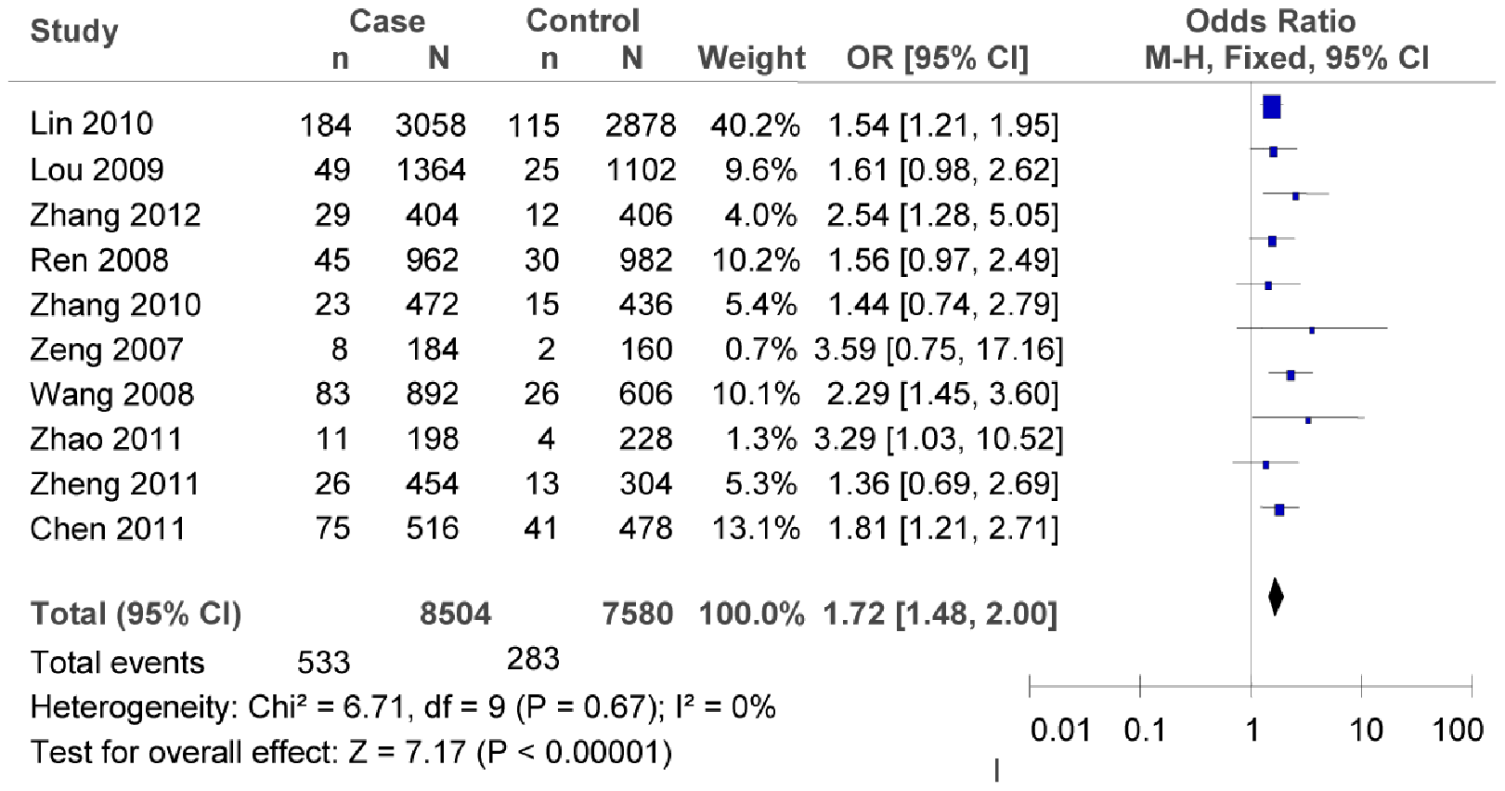
Forest plot of the association between rs7903146C/T polymorphism and T2DM risk (sensitivity analysis: T vs. C). n indicates the total number of T allele, and N indicates the total number of T allele plus C allele.

**Figure 14:**
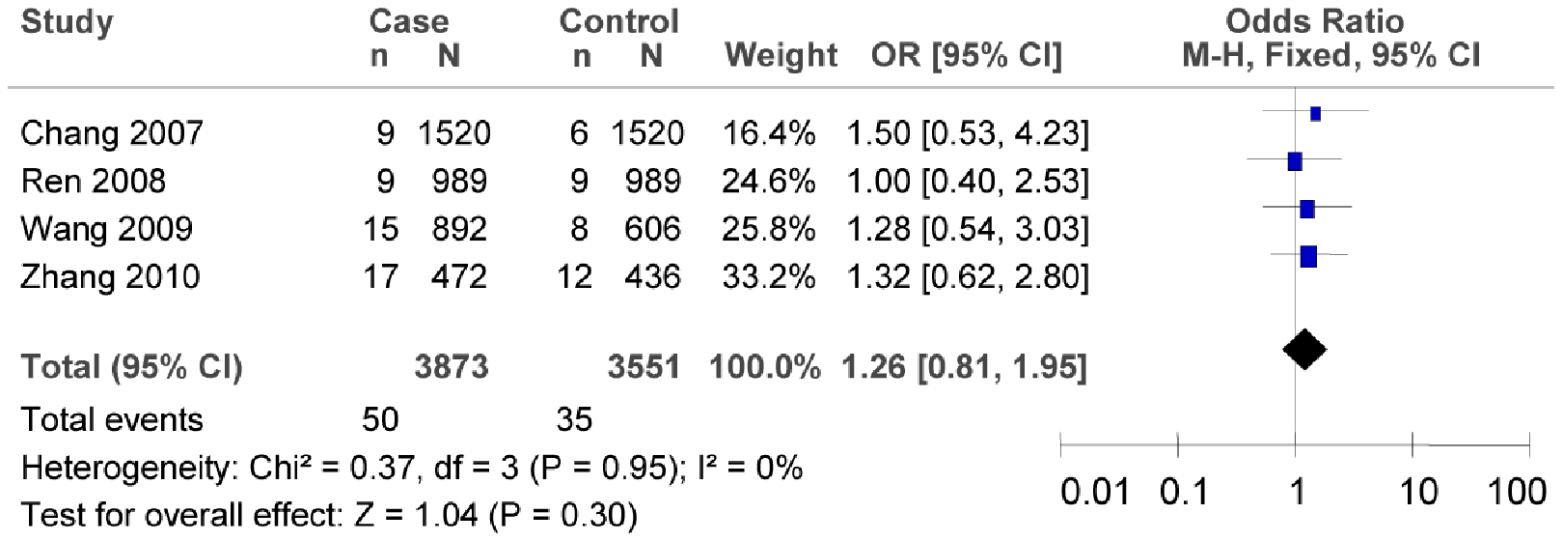
Forest plot of the association between rs12255372G/T polymorphism and T2DM risk (sensitivity analysis: T vs. G). n indicates the total number of T allele, and N indicates the total number of T allele plus G allele.

The sensitivity analysis of the studies on rs7903146 showed no heterogeneity, if omitting the studies by Chang [18], Ng [29], and Wen [32] from the total studies (as shown in Fig. 13, heterogeneity test *χ* ^2^ = 6.71, *P* = 0.67, *I^2^* = 0%). For this reason, we evaluated the association between rs7903146C/T polymorphism and T2DM risk by the fixed-effect model, and the interaction was confirmed (as shown in Fig. 13, T vs. C: *OR* = 1.72, 95% *CI* = 1.48–2.00, *P*<0.00001). For the sensitivity analysis of rs12255372, if omitting the study of Fan [15], the association between rs12255372 polymorphism and T2DM risk was not found (as shown in Fig. 14, T vs. G: *OR* = 1.26, 95%*CI* = 0.81–1.95, *P* = 0.30; heterogeneity test *χ* ^2^ = 0.37, *P* = 0.95, *I^2^* = 0%).

### Publication Bias

**Figure 15:**
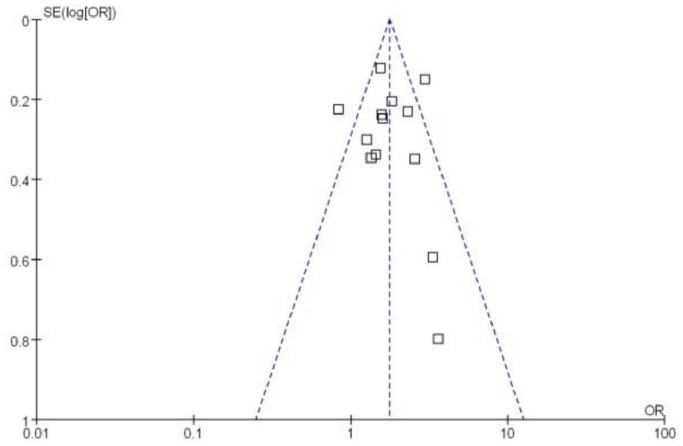
Funnel plots analysis to detect publication bias (T vs. C of rs7903146C/T polymorphism). Each point represents an independent study for the indicated association.Tables.

The shape of the funnel plots on the studies of rs7903146C/T polymorphism was symmetrical, suggesting that there was no evidence of publication bias for rs7903146C/T polymorphism (as shown in Figure 15). We did not make funnel plots for the other two single nucleotide polymorphisms (SNPs) due to the limited studies on rs12255372G/T and rs290487T/C.

## Discussion

It has been widely accepted that TCF7L2 gene is associate with T2DM risk in different ethnic groups [5], [9]–[10], [47]–[49]. Till now, no consistent results in Chinese Han population have been obtained. Chen [14], Lin [16], and Zhao [17] confirmed that there was association between the rs7903146 variant of TCF7L2 and T2DM risk in Chinese Han population. On the other hand, Chang [18], Ng [29], and Zheng [34] presented contrary conclusion. For resolving the conflict of all these studies, we employed a meta-analysis method to improve statistical power by pooling the related samples.

The study by Lou [50] indicated that four SNPs of TCF7L2 (rs7903146, rs12255372, rs11196205, and rs290487) were associated with T2DM risk in East Asian. Due to limited amounts of studies on SNPs of TCF7L2 in Chinese Han population, only three SNPs (rs7903146C/T, rs12255372G/T, and rs290487T/C) were analyzed in our meta-analysis. Our analysis indicated that rs7903146C/T polymorphism was significantly associated with T2DM risk in Chinese Han population, which is consistent with the studies in Europe [51]–[52] and East Asian [50]. The T allele at rs7903146 appeared to be one of the genetic risk factors for susceptibility to T2DM. On the other hand, we did not found any evidence that the association between T2DM risk and the other two SNPs (rs12255372G/T and rs290487T/C). It should be noted that this result is not consistent with the studies in Europe [51]–[52] and East Asian [50]. These discrepancies can be attributed to the difference between genetic backgrounds of ethnic and population substructure. Another possible explanation is that a lower risk allele frequency in Chinese Han population would be unlinked to the T2DM in spite of apparent association at the markers [53].

Compared with Europe population, there is a rather small genetic diversity in Chinese Han population [54]. Despite all that, Pritchard [55] illustrated that small diversity may be sufficient to lead to an inflated rate of false-positive results. In other words, the intricate substructure of Han Chinese may cause spurious association between polymorphism and T2DM risk. Xu [28] showed that there was the greatest genetic differentiation of Chinese Han population between the northern Han Chinese (NHC) and the southern Han Chinese (SHC) based on the genetic boundary of Yangtze River. Therefore, it is necessary to evaluate the effect of population substructure on the association between SNP of TCF7L2 and T2DM risk. A subgroup meta-analysis was utilized to explore the effect of the population substructure on the overall estimation of the association. Our analysis indicated that there was no association between SHC and NHC. On the other hand, the analysis result also reflected the heterogeneity among the included studies by heterogeneity test. We found that this heterogeneity of rs7903146 resulted from the studies by Chang [18], Ng [29], and Wen [32], while the heterogeneity of rs12255372 was attributed to the study by Fan [15]. As for rs7903146, the populations of the involved three studies resided in metropolis of Taiwan, HongKong and Shanghai, respectively. The residents in these bigalopolis emigrated from the different areas of China, and thus should not be regarded as one single homogenous population owing to historical immigrations, complex ancestries, population movements, and recent intermarriages with other ethnic groups in the three metropolises [28]. For this reason, our analysis may be affected by the intricate substructure in Han Chinese. Therefore, this is a feasible explanation for that the heterogeneity was observed from the study in the metropolis of Shanghai, but not found from the neighboring province of Jiangsu, despite both populations being SHC. With regard to rs12255372, Fan and his colleagues [15] illustrated that there was a significant association of rs12255372 with T2DM risk. This result was conflicted with other results [18]–[19], [31]. We considered this conflict may result from small samples, relatively low comparability between the cases and controls, difference in genotyping method, substructure of population, and some other unknown factors.

Besides the above mentioned population substructure, other confounder factors such as age, sex, BMI, environment, and ethnic may affect the study results of T2DM, since it is a complex hereditary disease. However, among all the studies included in our meta-analysis, only Lin [16], Chang [18], and Wang [31] demonstrated that the association between rs7903146C/T polymorphism and T2DM risk remained significant after adjustment for the combined confounders of age, sex, and BMI. This suggested that their effects on diabetes were not primarily mediated through adiposity [18]. Given most of the cases were not comparable with the controls on the age, sex, and BMI, it would be perfect to consider these factors in our meta-analysis. However, this was rarely achieved due to insufficient raw data. In spite of that, we employed subgroup analysis to evaluate the effect of some confounders like age, sex, and BMI on the pooled OR. The result indicated that the association of rs290487 with T2DM was influenced by BMI, whereas neither age nor sex affected the association of rs7903146 and rs290487 with T2DM.

The advantages of our meta-analysis can be summarized as follows. First of all, to the best of our knowledge, this is the most comprehensive meta-analysis for the association between TCF7L2 polymorphism (rs7903146C/T, rs12255372G/T and rs290487T/C) and T2DM risk in Chinese Han populations. The protocol of this meta-analysis has been well designed primitively by using explicit methods and criteria for study selection, data extraction, and data analysis. Perfect searching strategy based on computer-assisted search together with manual search has been applied to include eligible studies as many as possible. Finally, the quality of included studies in our meta-analysis is relatively satisfactory. Since the number of studies and subjects included in our meta-analysis were relatively small, however, the funnel plots could not be made in the two SNPs (rs12255372G/T and rs290487T/C), which could not avoid possible publication bias in our analysis.

In conclusion, this meta-analysis indicated that, in Chinese Han population, the rs7903146C/T polymorphism of TCF7L2 gene was associated with T2DM risk, while the polymorphisms of rs12255372G/T and rs290487T/C were not**.** We expect that the case-control studies with large and family-based samples had better be carried out in the future to provide sufficient data for better meta-analysis, considering low frequency of TCF7L2 gene, a variety of confounders, and two substructures in Chinese Han.

## Supporting Information

Table S1 **The PRISMA checklist for this meta-analysis.**
(DOC)

## References

[1] van Dieren S, Beulens JW, van der Schouw YT, Grobbee DE, Nealb B (2010) The global burden of diabetes and its complications: an emerging pandemic. Eur J Prev Cardiol (Suppl 1): 3–8. 10.1097/01.hjr.0000368191.86614.5a 20489418

[2] Shaw JE, Sicree RA, Zimmet PZ (2009) Global estimates of the prevalence of diabetes for 2010 and 2030. Diabetes Res Clin Pract 87: 4–14. 19896746 10.1016/j.diabres.2009.10.007

[3] Hansen L, Pedersen O (2005) Genetics of type 2 diabetes mellitus: status and perspectives. Diabetes Obes Metab 7: 122–135. 15715885 10.1111/j.1463-1326.2004.00396.x

[4] Grant SF, Thorleifsson G, Reynisdottir I, Benediktsson R, Manolescu A, et al (2006) Variant of transcription factor 7-like 2 (TCF7L2) gene confers risk of type 2 diabetes. Nat Genet 38: 320–323. 16415884 10.1038/ng1732

[5] Cauchi S, Meyre D, Dina C, Choquet H, Samson C, et al (2006) Transcription factor TCF7L2 genetic study in the French population: expression in human β-cells and adipose tissue and strong association with type 2 diabetes. Diabetes 55: 2903–2908. 17003360 10.2337/db06-0474

[6] Damcott CM, Pollin TI, Reinhart LJ, Ott SH, Shen H, et al (2006) Polymorphisms in the transcription factor 7- Like 2 (TCF7L2) gene are associated with type 2 diabetes in the Amish: Replication and evidence for a role in both insulin secretion and insulin resistance. Diabetes 55: 2654–2659. 16936218 10.2337/db06-0338

[7] Groves CJ, Zeggini E, Minton J, Frayling TM, Weedon MN, et al (2006) Association analysis of 6,736 U.K. subjects provides replication and confirms TCF7L2 as a type 2 diabetes susceptibility gene with a substantial effect on individual risk. Diabetes 55: 2640–2644. 16936215 10.2337/db06-0355

[8] Guinan KJ (2012) Worldwide distribution of type II diabetes-associated TCF7L2 SNPs: Evidence for stratification in Europe. Biochem Genet 50: 159–179. 21898192 10.1007/s10528-011-9456-2

[9] Lyssenko V, Lupi R, Marchetti P, Guerra SD, Orho-Melander M, et al (2007) Mechanisms by which common variants in the TCF7L2 gene increase risk of type 2 diabetes. J Clini Invest 117: 2155–2163. 10.1172/JCI30706 PMC1934596 17671651

[10] Potapov VA, Shamkhalova MN, Smetanina SA, Bel’chikova LN, Suplotova LA, et al (2010) Polymorphic markers TCF7L2 rs12255372 and SLC30A8 rs13266634 confer susceptibility to type 2 diabetes in a Russian population. Genetika 46: 1123–1131. 20873210

[11] Helgason A, Pálsson S, Thorleifsson G, Grant SF, Emilsson V, et al (2007) Refining the impact of TCF7L2 gene variants on type 2 diabetes and adaptive evolution. Nat Genet 39: 218–225. 17206141 10.1038/ng1960

[12] Lehman DM, Hunt KJ, Leach RJ, Hamlington J, Arya R, et al (2007) Haplotypes of transcription factor 7-like 2 (TCF7L2) gene and its upstream region are associated with type 2 diabetes and age of onset in Mexican Americans. Diabetes 56: 389–393. 17259383 10.2337/db06-0860

[13] Chandak GR, Janipalli CS, Bhaskar S, Kulkarni SR, Mohankrishna P, et al (2007) Common variants in the TCF7L2 gene are strongly associated with type 2 diabetes mellitus in the Indian population. Diabetologia 50: 63–67. 17093941 10.1007/s00125-006-0502-2

[14] Chen GY, Zhang JH (2011) Relationship between TCF7L2 polymorphism with Type 2 Diabetes and related factors. Journal of Xianning University (Medical Sciences) 25: 103–106. [Article in Chinese].

[15] Fan XW (2010) The association study of TCF7L2, AKT2, FOXO1 gene polymorphisms with type 2 diabetes in Tianjin Han population and families. Master thesis. [Article in Chinese].

[16] Lin Y, Li PQ, Cai L, Zhang B, Tang X, et al (2010) Association study of genetic variants in eight genes/loci with type 2 diabetes in a Han Chinese population. BMC Medical Genetics 11: 1–8. 20550665 10.1186/1471-2350-11-97 PMC2894791

[17] Zhao T, Zhao SH, Wang YG, Liu SG, Liu J, et al.. (2011) The association of transcription factor 7-like 2 polymorphism with the early phase function of β-cell in type 2 diabetes mellitus. Med J Qilu 26: 417–420. [Article in Chinese].

[18] Chang YC, Chang TJ, Jiang YD, Kuo SS, Lee KC, et al (2007) Association study of the genetic polymorphisms of the transcription factor 7-like 2 (TCF7L2) gene and type 2 diabetes in the Chinese population. Diabetes 56: 2631–2637. 17579206 10.2337/db07-0421

[19] Zhang L (2010) Replication study for the association of TCF7L2 rs12255372 and rs7903146 with susceptibility to type 2 diabetes in a Chinese Han population. Master thesis. [Article in Chinese].

[20] Xu SH, Yin XY, Li SL, Jin WF, Lou HY, et al (2009) Genomic dissection of population substructure of Han Chinese and its implication in association studies. Am J Hum Genet 85: 762–774. 19944404 10.1016/j.ajhg.2009.10.015 PMC2790582

[21] Thakkinstian A, McElduff P, D’Este C, Duffy D, Attia J (2005) A method for meta-analysis of molecular association studies. Stat Med 24: 1291–1306. 15568190 10.1002/sim.2010

[22] Moher D, Liberati A, Tetzlaff J, Altman DG (2010) Preferred reporting items for systematic reviews and meta-analyses: the PRISMA statement. Int J Surg 8: 336–341. 20171303 10.1016/j.ijsu.2010.02.007

[23] Wells G, Shea B, O ’Connell D, Peterson J, Welch V, et al (2011) The Newcastle-Ottawa Scale (NOS) for assessing the quality of case-control studies in meta-analyses. European Journal of Epidemiology 25: 603–605. 10.1007/s10654-010-9491-z 20652370

[24] Lau J, Ioannidis JP, Schmid CH (1997) Quantitative synthesis in systematic reviews. Ann Intern Med 127: 820–826. 9382404 10.7326/0003-4819-127-9-199711010-00008

[25] Mantel N, Haenszel W (1959) Statistical aspects of the analysis of data from retrospective studies of disease. J Natl Cancer Inst 22: 719–748. 13655060

[26] DerSimonian R, Laird N (1986) Meta-analysis in clinical trials. Control Clin Trials 7: 177–188. 3802833 10.1016/0197-2456(86)90046-2

[27] Higgins JP, Thompson SG, Deeks JJ, Altman DG (2003) Measuring inconsistency in meta-analyses. BMJ 327: 557–560. 12958120 10.1136/bmj.327.7414.557 PMC192859

[28] Xu SH, Yin XY, Li SL, Jin WF, Lou HY, et al (2009) Genomic dissection of population substructure of Han Chinese and its implication in association studies. Am. J. Hum. Genet 85: 762–774. 10.1016/j.ajhg.2009.10.015 PMC2790582 19944404

[29] Ng MC, Tam CH, Lam VK, So WY, Ma RC (2007) Replication and identification of novel variants at TCF7L2 associated with type 2 diabetes in Hong Kong Chinese. J Clin Endocr Metab 92: 3733–3737. 17609304 10.1210/jc.2007-0849

[30] Ren Q, Han X Y, Wang F, Zhang XY, Han LC, et al (2008) Exon sequencing and association analysis of polymorphisms in TCF7L2 with type 2 diabetes in a Chinese population. Diabetologia 51: 1146–1152. 18493736 10.1007/s00125-008-1039-3

[31] Wang ZH (2008) Association of polymorphisms in transcription factor 7-like 2 (TCF7L2) gene and solute carrier family30, member8 with type 2 diabetes. Doctoral thesis. [Article in Chinese].

[32] Wen J, Rönn T, Olsson A, Yang Z, Lu B, et al (2010) Investigation of type 2 diabetes risk alleles support CDKN2A/B, CDKAL1, and TCF7L2 as susceptibility genes in a Han Chinese Cohort. PLoS ONE 5: e9153. 20161779 10.1371/journal.pone.0009153 PMC2818850

[33] Zeng QC (2007) Study of rs7903146 polymorphism in the transcription factor 7-like 2 (TCF7L2) gene in type 2 diabetes mellitus of Chinese in Chengdu area. Master thesis. [Article in Chinese].

[34] Zheng XY, Ren W, Zhang SH, Liu JJ, Li SF, et al (2012) Association of type 2 diabetes susceptibility genes (TCF7L2, SLC30A8, PCSK1 and PCSK2) and proinsulin conversion in a Chinese population. Mol Biol Rep 39: 17–23. 21437630 10.1007/s11033-011-0705-6

[35] Lou QL, Bian RW, Xie CY, Gu LB, Xia H, et al.. (2009) Association between the single nucleotide polymorphism of the transcription factor 7-like 2 gene and the genetic susceptibility of type 2 diabetes in Han Chinese population. Chin J Diabetes 17: 895–898. [Article in Chinese].

[36] Zhang XL, Wang YX, L W, B C, Ji HM. (2012) Relationship between rs7903146-T/C polymorphism of TCF7L2 Gene and Type 2 Diabetes in Shenyang Population. Chin J Misdiagn 12: 255–256. [Article in Chinese].

[37] Zou YL, Gao JM, Xu Y, Tang H, Peng ZG, et al.. (2009) Relationship between rs290487- T/C Polymorphism of TCF7L2 Gene and Type 2 Diabetes in Kunming Han Population. China Modern Doctor 47: 11–12. [Article in Chinese].

[38] Zhang Y, Rong HQ, Wang H, Ding HF, Li YP, et al.. (2009) Relationship between rs290487- T/C Polymorphism of TCF7L2 Gene and Type 2 Diabetes. Journal of Practical diabetology 5: 27–29. [Article in Chinese].

[39] Zhang Y. (2008) Association of polymorphisms in TCF7L2 gene with type 2 diabetes in Chinese Han population. Master thesis. [Article in Chinese].

[40] Qiao H, Zhang XY, Zhao XD, Zhao YL, Xu LD, et al (2012) Genetic variants of TCF7L2 are associated with type 2 diabetes in a northeastern Chinese population. Gene 495: 115–119. 22245614 10.1016/j.gene.2011.12.055

[41] Yu M, Xu XJ, Yin JY, Wu J, Chen X, et al (2010) KCNJ11 Lys23Glu and TCF7L2 rs290487(C/T) polymorphisms affect therapeutic efficacy of repaglinide in Chinese patients with type 2 diabetes. Nature publishing group 87: 330–335. 10.1038/clpt.2009.242 20054294

[42] Zhu H, Wang YM, Xu M, Xu ZS. Biochip techniques applied to detect SNP rs290487 in TCF7L2 gene for rapid screening of diabetes susceptibility. Acta Univ. Med. Anhui 46: 20–24. [Article in Chinese].

[43] Wang X, Luo X, Wang Y, Zhang MF, Mao XM, et al.. (2010) The correlation study of TCF7L2 and type 2 diabetes mellitus in Uygurs. Journal of Xinjiang Medical University 33: 1162–1164. [Article in Chinese].

[44] Wang ZH, Zhang SH, Wang ZC, Gong LL, Li R, et al.. (2009) Association of polymorphisms in transcription factor 7-like 2 (TCF7L2) gene with type 2 diabetes in Chinese Han population. Chin J Endocrnol Metab 25: 139–143. [Article in Chinese].

[45] Fan XW, Liu DM, Sun Y, Zhang J, Shi XW (2009) Study on polymorphisms of TCF7L2 gene association with type 2 diabetes based on DHPLC detection. Tianjin Med J 37: 835–838. [Article in Chinese].

[46] Zheng XY, Ren W, Zhang SH, Liu JJ, Li SF, et al.. (2011) Correlation between single nucleotide polymorphisms of rs7903146 and rs11196218 at TCF7L2 gene and the early phase insulin secretion of newly diagnosed patients with type 2 diabetes. Med J Chin PLA 36: 269–272. [Article in Chinese].

[47] Hayashi T, Iwamoto Y, Kaku K, Hirose H, Maeda S (2007) Replication study for the association of TCF7L2 with susceptibility to type 2 diabetes in a Japanese population. Diabetologia 50: 980–984. 17340123 10.1007/s00125-007-0618-z

[48] Miyake K, Horikawa Y, Hara K, Yasuda K, Osawa H, et al (2008) Association of TCF7L2 polymorphisms with susceptibility to type 2 diabetes in 4,087 Japanese subjects. J Hum Genet 53: 174–180. 18097733 10.1007/s10038-007-0231-5

[49] Palizban A, Nikpour M, Salehi R, Maracy MR (2012) Association of a common variant in TCF7L2 gene with type 2 diabetes mellitus in a Persian population. Clin Exp Med 12: 115–119. 21678030 10.1007/s10238-011-0144-7

[50] Lou YY, Wang HY, Han XY, Ren Q, Wang F, et al (2009) Meta-analysis of the association between SNPs in TCF7L2 and type 2 diabetes in East Asian population. diabetes research and clinical practice 85: 139–146. 19482368 10.1016/j.diabres.2009.04.024

[51] Scott LJ, Bonnycastle LL, Willer CJ, Sprau AG, Jackson AU, et al (2006) Association of Transcription Factor 7-Like 2 (TCF7L2) Variants With Type 2 Diabetes in a Finnish Sample. Diabetes 55: 2649–2653. 16936217 10.2337/db06-0341

[52] van Vliet-Ostaptchouk JV, Shiri-Sverdlov R, Zhernakova A, Strengman E, van Haeften TW, et al (2007) Association of variants of transcription factor 7- like 2 (TCF7L2) with susceptibility to type 2 diabetes in the Dutch Breda cohort. Diabetologia 50: 59–62. 17031610 10.1007/s00125-006-0477-z

[53] International HapMap Consortium (2005) A haplotype map of the human genome. Nature 437: 1299–1320. 16255080 10.1038/nature04226 PMC1880871

[54] Xu S, Jin L (2008) A Genome-wide Analysis of Admixture in Uyghurs and a High-Density Admixture Map for Disease-Gene Discovery. Am. J. Hum. Genet 83: 322–336. 10.1016/j.ajhg.2008.08.001 PMC2556439 18760393

[55] Pritchard JK, Donnelly P (2001) Case–Control Studies of Association in Structured or Admixed Populations. Theor. Popul. Biol 60: 227–237. 10.1006/tpbi.2001.1543 11855957

